# Simulation Model of Wind and Wave-Induced Doppler Shifts for Multi-Band Radars and Its Application in SAR-Based Ocean Current Inversion

**DOI:** 10.3390/s26041343

**Published:** 2026-02-19

**Authors:** Zhenyong Guan, Yubin Zhang, Xiaoliang Chu

**Affiliations:** 1 College of Information Science and Engineering, Ocean University of China, Qingdao 266100, China; guanzhenyong@stu.ouc.edu.cn (Z.G.); xlchu@ouc.edu.cn (X.C.); 2 Engineering Research Center of Advanced Marine Physical Instruments and Equipment, Ministry of Education, Qingdao 266100, China

**Keywords:** wind and wave-induced Doppler shift (WDS), ocean surface current fields retrieval, synthetic aperture radar (SAR), multiple radar bands

## Abstract

**Highlights:**

**What are the main findings?**

- Developed a multi-band applicable simulation model for wind and wave-induced Doppler shift, which shows correlation coefficients of 0.97, 0.93, and 0.98 relative to the CDOP, KaDOP and KuMOD models, respectively.
- Using the proposed model, ocean currents retrieved based on Sentinel-1 ocean (OCN) data achieve a mean deviation (MD) of −0.04 m/s, a mean absolute error (MAE) of 0.26 m/s, and a root-mean-square error (RMSE) of 0.32 m/s against high-frequency (HF) radar measurements.

**What are the implications of the main findings?**

- The proposed model can be further applied to retrieve ocean currents based on radar data of different frequency bands, providing an effective approach for removing the wind and wave-induced Doppler shift across varying bands.

**Abstract:**

The wind and wave-induced Doppler shift (WDS) significantly affects the accuracy of ocean surface current fields retrieved from synthetic aperture radar (SAR). Understanding how different factors affect WDS is therefore essential for improving current inversion accuracy. Existing studies have predominantly focused on single-band WDS, mainly in the C-band, while investigations across other radar bands remain limited. In this study, we simulate the dynamic ocean surface height field and velocity field, and the radar backscatter from the ocean surface that includes the effect of breaking waves. Based on the Doppler shift theory of ocean surface motion proposed by Chapron, we develop a WDS simulation model with potential applicability to multiple radar bands. The performance of the model is verified by comparing its results with those from the CDOP, KaDOP and KuMOD models. The correlation coefficient between the proposed model and the CDOP model reaches 0.97, with mean deviation (MD), mean absolute error (MAE), and root-mean-square error (RMSE) not exceeding −2.07 Hz, 3.35 Hz, and 4.49 Hz, respectively. For comparisons with the KaDOP model, the correlation coefficient is 0.93, and the MD, MAE, and RMSE are within −21.23 Hz, 42.37 Hz, and 52.20 Hz. For comparisons with the KuMOD model, the correlation coefficient is 0.98, and the MD, MAE, and RMSE are within −2.60 Hz, 7.13 Hz, and 9.08 Hz. These results demonstrate that the proposed model can effectively predict the WDS for both C-, Ka-, and Ku-band radar returns. Furthermore, we investigate the impacts of radar parameters, including frequency band, polarization, and incidence angle, as well as wind field forcing on WDS, showing the model’s applicability across multiple radar bands. Finally, the proposed model is applied to current retrieval using Sentinel-1 ocean (OCN) data, and the inversion accuracy is assessed against collocated high-frequency (HF) radar observations. The MD, MAE, and RMSE of the current retrieval using the proposed model are −0.04 m/s, 0.26 m/s, and 0.32 m/s, which are close to those from the CDOP-based retrieval (MD, MAE, and RMSE of −0.02 m/s, 0.25 m/s, and 0.30 m/s). These results demonstrate that the proposed model performs well in ocean surface current inversion and shows potential for further application to ocean current retrieval based on radar data across different frequency bands.

## 1. Introduction

An ocean current is relatively stable movements of seawater over large spatial scales and is an important component of ocean motion. It significantly influences marine ecosystems, geological processes, and climate change [1]. The detection and acquisition of ocean current information have important scientific and practical value. Remote sensing has emerged as a key method for observing ocean surface current fields due to its advantages of wide coverage [2] and high accuracy [3,4,5]. The main techniques include altimeters [6], high-frequency radar [7], Doppler scatterometer [8], and synthetic aperture radar (SAR) [9,10]. Among these, SAR can provide all-time, all-weather, wide-coverage, and high-accuracy measurements, making it a promising approach for observing ocean surface current fields [11].

The main SAR-based methods for measuring ocean surface current fields include the Along-Track Interferometric method (ATI) [12] and Doppler Centroid Analysis (DCA) [13]. The ATI method retrieves ocean surface currents from the phase difference between two complex images acquired by a pair of antennas. It provides high accuracy and high resolution, but the strict requirements for dual antennas limit its applications [14]. The DCA method retrieves ocean surface currents by using the relationship between the Doppler shift caused by ocean surface motion and radial velocity [15]. Although this method offers relatively low resolution, it requires only a single antenna and benefits from richer data sources, making it more widely applicable. However, whether using the ATI or DCA method, the retrieved ocean surface velocities result from the combined effects of wind and waves as well as ocean currents. The motion caused by wind and waves has an important influence on current retrieval [16,17]. Therefore, accurately estimating and removing the effects of wind and waves on ocean surface Doppler velocity is a key step for precise current inversion [18]. Many studies have focused on removing the influence of wind and waves in ocean surface current retrieval. These studies can be categorized into two types: empirical models based on neural networks [11,13,19,20,21,22,23,24,25] and theoretical models based on ocean surface scattering mechanisms [26,27,28,29,30,31].

In terms of empirical models, Mouche et al. developed the empirical geophysical model CDOP based on a three-layer neural network [19]. This model estimates the Doppler shift for a given polarization when the wind speed, wind direction, and SAR incidence angle are known, but it does not account for the influence of swell on the Doppler shift. Moiseev et al. proposed the CDOP3S model, which takes both wind waves and swell into account [13]; however, this model suffered from overfitting. Therefore, the team further developed an improved CDOP3SX model [20] and the CDOP3SiX model [20], which can separately calculate the Doppler shifts caused by wind waves and swell. Yurovsky et al. developed a semi-empirical model, KaDOP [21], based on Ka-band radar data from the Black Sea research platform, to estimate the wind and wave-induced Doppler shift (WDS) in the Ka-band. Cui et al. developed a semi-theoretical model, KuMOD (Ku-band Doppler Shift Model) [22], based on Ku-band empirical geophysical model functions, to estimate the WDS in the Ku band. Recently, Yang et al. constructed four empirical geophysical models, CDOP-Yn [23], to estimate the effects of different wind and wave conditions on SAR Doppler shift and achieved relatively high-accuracy current retrieval using these models. Bai et al. developed the OCN-CRM model [11], which directly retrieves ocean surface currents from Sentinel-1-measured data using neural networks, showing robust improvements in current retrieval accuracy compared with traditional methods. Sun et al. developed the OSCNet model, which integrates multiple data sources with neural networks and effectively improves the accuracy of SAR-based current retrieval [24]. The aforementioned empirical models based on neural networks have greatly advanced the removal of wind and wave effects during current retrieval and improved the accuracy of retrieved currents. However, these empirical models are not only heavily reliant on specific satellite platforms—limiting their universality [25]—but also lack the capability to elucidate the deeper physical mechanisms governing Doppler shifts.

In terms of theoretical models, Chapron et al. analyzed Doppler shift data obtained from Envisat ASAR and found a strong correlation between Doppler shift and ocean surface wind field [26]. They also established a simplified Doppler velocity model for wind and current fields [27]. Kudryavtsev et al. developed the radar imaging model (RIM), which can calculate Bragg scattering and breaking wave scattering from the ocean surface [28]. Johannessen et al. extended the application of the RIM in observing ocean surface current fields and proposed the DopRIM (Doppler Radar Imaging Model) model [29], which can be used to calculate the Doppler velocity of different components of the ocean surface, including orbital velocities of large-scale waves, phase velocities of Bragg waves, and velocities of breaking waves. Kudryavtsev et al. proposed a semi-empirical and semi-theoretical model, DPDOP, based on C-SARMOD2 [29]. Subsequently, Fan et al. simplified the model and used it to achieve relatively accurate retrieval of ocean surface current velocity [30]. In addition, Fan et al. proposed a new method for reconstructing ocean surface current vectors based on Doppler shift data from Sentinel-1 SAR [31], which shows good potential in reconstructing ocean surface vector fields. Theoretical models can be applied to multiple radar bands and can reveal the physical mechanisms by which wind and waves affect Doppler shift. However, the practical utility of these models is often constrained by the complexity inherent in their application, stemming from the numerous physical approximations necessary during their derivation.

As mentioned above, empirical models cannot provide a deep analysis of physical mechanisms and have limited universality, while directly applying theoretical models to ocean surface current retrieval is very complex. In the field of ocean remote sensing, simulation has become an important research tool because it allows flexible parameter settings, can model various ocean surface scenarios, and does not rely on measured data. Therefore, this study developed a universal multi-band WDS simulation model based on simulations of ocean surface height, water particle velocity, radar backscatter, and Doppler shift. Using this model, we then investigated the effects of radar parameters and sea conditions on WDS across multiple bands. In addition, based on Sentinel-1 ocean (OCN) data, we implemented and validated ocean surface current retrieval using the proposed model.

The structure of this paper is organized as follows. Section 2 introduces the theory and simulation methods for calculating WDS. Section 3 presents the simulation results and validation, showing the C-band simulation results and comparisons with the CDOP model. Section 4 discusses the effects of different factors on WDS across multiple bands and applies the proposed model to ocean surface current retrieval. Section 5 provides a summary.

## 2. Materials and Methods

In this study, we simulated the ocean surface height field, the velocity of water particles at the surface—including wave orbital velocity, Bragg wave phase velocity, wind drift velocity, and breaking wave velocity—and the radar backscatter from the ocean surface. Following the Doppler shift theory of sea surface motion proposed by Chapron [27], we further simulated the wind and wave-induced Doppler shift (WDS). The theoretical methods employed in these simulations are detailed in the following sections.

### 2.1. Doppler Shift Theory of Ocean Surface Motion

The Doppler shift $f_{Dc}$ measured by spaceborne SAR consists of multiple components [13,27], which can be expressed as follows

(1) $$f_{Dc}=f_{sat}+f_{elec}+f_{phys}+∆f$$

where $f_{sat}$ and $f_{elec}$ represent the Doppler shifts caused by the relative motion between the satellite and the Earth’s surface, and by platform or antenna perturbations, respectively. $f_{phys}$ is the Doppler shift jointly induced by wind and waves as well as ocean currents. ∆f denotes the residual Doppler shift error. Reliable methods already exist to remove the effects of $f_{sat}$ and $f_{elec}$ [23,27,32]. $f_{phys}$ can be obtained by performing a weighted average of the velocity components of ocean surface facets within the radar incidence plane, using the radar backscatter cross section as the weight [27]

(2) $$\frac{\pi f_{phys}\left(\theta_{\omega },U_{10},\theta ,pol\right)}{k_{e}}=-\frac{\bar{\left(usin\theta -\omega cos\theta \right)\sigma^{0}\left(\theta +∆\theta \right)}}{\bar{\sigma^{0}\left(\theta +∆\theta \right)}}$$

where $k_{e}$ denotes the wavenumber of the incident radar electromagnetic wave, $\sigma^{0}$ is the normalized radar backscattering cross section (NRCS), and $\theta_{\omega },U_{10},\theta ,pol$ represent the wind direction angle (i.e., the angle between wind direction and radar look direction), wind speed at 10 m above the sea surface, radar incidence angle, and polarization mode, respectively. ∆θ represents the local correction of the radar incidence angle θ caused by the tilt of the ocean surface facet. u and ω are the horizontal and vertical velocity components of the ocean surface facet within the radar incidence plane, respectively.

Wind and wave-induced velocities on the ocean surface include wave orbital velocity, Bragg wave phase velocity, wind drift velocity, and breaking wave velocity. Among these components, the contributions of the Bragg wave phase velocity and the breaking wave velocity are weighted according to the respective proportions of Bragg scattering and wave breaking scattering in the total radar scattering [25,33,34]. Therefore, the terms u and ω in (2) can be expressed as

(3) $$u=v_{ox}cos\varphi_{SAR}+v_{oy}sin\varphi_{SAR}+P_{b}v_{b}\left(\theta_{w}\right)+P_{wb}v_{wb}\left(\theta_{w}\right)+v_{drift}\left(\theta_{w}\right)$$

(4) $$\omega =v_{oz}$$

where $v_{ox}$, $v_{oy}$, and $v_{oz}$ are the components of the wave orbital velocity in different directions, $\varphi_{SAR}$ is the angle between the radar look direction and the x axis, and $\theta_{\omega }$ is the angle between the wind direction and the radar look direction. $v_{b}\left(\theta_{w}\right)$, $v_{wb}\left(\theta_{w}\right)$, and $v_{drift}\left(\theta_{w}\right)$ are the Bragg wave phase velocity, breaking wave velocity, and wind drift velocity along the radar look direction, respectively. $P_{b}$ and $P_{wb}$ represent the proportions of Bragg scattering and wave breaking scattering in the total radar scattering, respectively.

### 2.2. Dynamic Ocean Surface Modeling Based on Potential Flow Theory

In this study, the dynamic ocean surface is modeled and simulated based on linear wave theory. The primary methods for linear ocean surface modeling include the linear superposition method and the linear filtering method [35,36]. Based on the ocean surface velocity potential, the ocean surface elevation field and the corresponding orbital velocity fields can be derived. Assuming seawater is incompressible and its motion is irrotational, the linear superposition method is adopted to construct the ocean surface elevation $\eta_{L}$ and the corresponding orbital velocity fields $v_{ox}$, $v_{oy}$, and $v_{oz}$, from the ocean surface velocity potential φ [37,38]

(5) $$\varphi \left(x,y,z,t\right)=g\sum_{i}\sum_{j}\frac{a_{ij}}{\omega_{i}}e^{k_{i}z}sin\left[k_{i}\left(xcos\theta_{j}+ysin\theta_{j}\right)-\omega_{i}t+\varepsilon_{ij}\right],$$

(6) $$\eta_{L}=-\frac{1}{g}\frac{\partial \varphi }{\partial t}{|}_{z=0},$$

(7) $$v_{ox}=\frac{\partial \varphi }{\partial x}{|}_{z=0},v_{oy}=\frac{\partial \varphi }{\partial y}{|}_{z=0},v_{oz}=\frac{\partial \varphi }{\partial z}{|}_{z=0}$$

where $\omega_{i}$, $k_{i}$, $\theta_{j}$, and $\varepsilon_{ij}$ represent the angular frequency, wave number, direction angle, and initial random phase of the ocean surface component waves, respectively. t and g denote time and gravitational acceleration, respectively. $\eta_{L}$ represents the ocean surface elevation, and z=0 represents the mean position of the ocean surface fluctuations. $a_{ij}$ is the amplitude term, which is usually calculated by the following equation [37,38]

(8) $$a_{ij}=\sqrt{2S\left(\omega_{i},\theta_{j}\right)\delta \omega_{i}\delta \theta_{j}}$$

where $\delta \omega_{i}$ and $\delta \theta_{j}$ are the sampling intervals of the angular frequency and the direction angle of the ocean surface component waves, respectively. $S\left(\omega_{i},\theta_{j}\right)$ is the two-dimensional directional frequency spectrum of the ocean surface. In this study, the two-dimensional PM spectrum [39] is selected to simulate the large-scale ocean surface. The relevant input parameters used in the dynamic sea surface simulation are shown in Table 1.

### 2.3. Radar Backscatter Modeling

At moderate incidence angles, the dominant scattering mechanism of the ocean surface is Bragg scattering. However, many studies have shown that scattering from breaking waves cannot be ignored [34,40]. Phillips suggests that ocean surface backscattering is the sum of contributions from Bragg scattering and breaking wave scattering [33,41]. In this study, the radar backscattering coefficient of the ocean surface is calculated by using the two-scale composite surface model (TSM) [42] combined with a breaking wave scattering model [41]

(9) $$\sigma^{0}=\sigma_{TSM}^{\prime }\left(1-q\right)+\sigma_{wb}q$$

where $\sigma^{0}$ is the total radar scattering coefficient, $\sigma_{TSM}^{\prime }$ is the ocean surface Bragg scattering coefficient that accounts for the modulation of large-scale waves, and $\sigma_{wb}$ is the breaking wave scattering coefficient. q represents the fraction of the ocean surface covered by breaking waves, which was obtained by Huang et al. through fitting to measured data [43]

(10) $$q=\left(11.12e^{0.063U_{19.5}}-16.24\right)\%,U_{19.5}⩾7m/s$$

where $U_{19.5}$ is the wind speed at a height of 19.5 m above the ocean surface.

Based on the TSM [42], the ocean surface Bragg scattering coefficient with tilt modulation can be expressed as

(11) $$\sigma_{TSM}^{HH}=8\pi {k_{e}}^{4}cos^{4}\theta_{loc}\left[S_{s}\left(-{\vec{k}}_{b}\right)+S_{s}\left({\vec{k}}_{b}\right)\right]{\left|g_{HH}cos^{2}\gamma +g_{VV}sin^{2}\gamma \right|}^{2}$$

(12) $$\sigma_{TSM}^{VV}=8\pi {k_{e}}^{4}cos^{4}\theta_{loc}\left[S_{s}\left(-{\vec{k}}_{b}\right)+S_{s}\left({\vec{k}}_{b}\right)\right]{\left|g_{HH}sin^{2}\gamma +g_{VV}cos^{2}\gamma \right|}^{2}$$

where $k_{e}$ is the wave number of the incident electromagnetic wave, and $S_{s}\left(k\right)$ is the small-scale wave spectrum of the ocean surface. ${\vec{k}}_{b}$ is the Bragg wave vector, which can be expressed as $2\left(\vec{k_{H}}+\vec{k_{Z}}\nabla \eta_{L}\right)$. ${\vec{k}}_{H}$ and ${\vec{k}}_{Z}$ are the horizontal and vertical components of the electromagnetic wave vector, respectively, and $\nabla \eta_{L}$ is the slope of long waves. γ is the approximate rotation angle between the incident electromagnetic wave and the facet normal. $g_{HH}$ and $g_{VV}$ are geometric scattering coefficients. The detailed expressions of γ, $g_{HH}$, and $g_{VV}$ can be found in Reference [42]. $\theta_{loc}$ is the local incidence angle, which can be expressed by the unit vector $\frac{{\vec{k}}_{e}}{k_{e}}$ of the incident electromagnetic wave vector and the unit normal vector $\vec{n}$ of the tilted facet modulated by long waves as

(13) $$cos\theta_{loc}=-\frac{{\vec{k}}_{e}\cdot \vec{n}}{k_{e}}$$

The distribution of small-scale ocean surface waves is affected by long-wave slopes. The local incidence angle $\theta_{loc}$ in (11) and (12), as well as ${\vec{k}}_{z}\nabla \eta_{L}$ in the Bragg wave vector ${\vec{k}}_{b}$, both reflect the effect of long-wave tilt modulation. In addition, the hydrodynamic modulation between long waves and short waves can cause variations in the local small-scale wave spectrum on the ocean surface. The hydrodynamic modulation function can be expressed as [44]

(14) $$T_{k}^{h}=-4.5\left|k\right|\omega \frac{\omega -j\mu }{\omega^{2}+\mu^{2}}sin^{2}\varphi_{a}$$

where k is the long-wave wave number, ω is the angular frequency, μ is the relaxation factor, and $\varphi_{a}$ is the angle between the wave propagation direction and the azimuth direction. By applying the double superposition model, the modulation function of the ocean surface over the SAR integration time $T_{SAR}$ can be obtained [45,46]

(15) $$f\left(x,y,t\right)=\sum_{i}\sum_{j}A_{ij}^{T}a_{ij}\left|T_{k}^{h}\right|cos\left[k_{i}\left(xcos\theta_{j}+ysin\theta_{j}\right)-\omega_{i}t+\varepsilon_{ij}+\phi_{R}\right]$$

where $\left|T_{k}^{h}\right|$ and $\phi_{R}$ are the modulus and phase of the hydrodynamic modulation function $T_{k}^{h}$, respectively. $A_{ij}^{T}$ is a function related to the radar integration time $T_{SAR}$, the wave number $k_{i}$, and other parameters, and it can be expressed as [45,46]

(16) $$A_{ij}^{T}=sinc\left(\frac{k_{x}∆x}{2}\right)sinc\left(\frac{k_{y}∆y}{2}\right)sinc\left(\frac{\omega_{i}T_{SAR}}{2}\right)$$

where $k_{x}$ and $k_{y}$ represent the components of the wave number of the ocean surface constituent waves in the x and y directions, which can be expressed as $k_{i}cos\theta_{j}$ and $k_{i}sin\theta_{j}$, respectively. ∆x and ∆y denote the discrete intervals of the ocean surface facets, and $T_{SAR}$ is the radar integration time.

Under the effects of tilt modulation and hydrodynamic modulation, the Bragg backscattering coefficient can be expressed as [45,46]

(17) $$\sigma_{TSM}^{\prime }=\sigma_{TSM}^{pp}\left[1+f\left(x,y,t\right)\right]$$

where $\sigma_{TSM}^{\prime }$ is the Bragg backscattering coefficient accounting for tilt and hydrodynamic modulation, and pp represents the polarization mode.

Wave breaking is a strong wave–wave interaction phenomenon. Kudryavtsev et al. [41] suggest that the scattering coefficient of breaking waves can be expressed as

(18) $$\sigma_{wb}=\left(\frac{sec^{2}\theta_{loc}}{s_{wb}^{2}}\right)exp\left(-\frac{tan^{2}\theta_{loc}}{s_{wb}^{2}}\right)+\frac{\varepsilon_{wb}}{s_{wb}^{2}}$$

where $s_{wb}$ is the root mean square of the roughness increased by the breaking wave area, $\varepsilon_{wb}$ is the ratio of the thickness to the length of the breaking elements, and according to experimental data from Unal and Masuko [47,48], $s_{wb}^{2}$ and $\varepsilon_{wb}$ are taken as 0.19 and 0.005, respectively.

### 2.4. Simulation of Various Velocity Components of Ocean Surface Motion

In SAR ocean current remote sensing, the Doppler signal of the detected sea surface echo represents the total Doppler signal generated by sea surface motion, of which the velocities associated with wind and waves on the ocean surface include wave orbital velocity, Bragg wave phase velocity, wind drift velocity, and breaking wave velocity. The wave orbital velocity can be obtained from (7) in Section 2.2, while the remaining velocity components need to be simulated.

#### 2.4.1. Bragg Phase Velocity and Wind Drift Velocity

The Bragg phase velocity detected by SAR is the vector sum of the Bragg phase velocities toward and away from the radar, that is, the net Bragg phase velocity $v_{b}$, which can be expressed as [49,50]

(19) $$v_{b}\left(\theta_{w}\right)=c\left(k_{b}\right)\left[\frac{G\left(\theta_{w}\right)-G\left(\theta_{w}+\pi \right)}{G\left(\theta_{w}\right)+G\left(\theta_{w}+\pi \right)}\right]$$

where $\theta_{\omega }$ is the angle between the wind direction and the radar look direction, $c\left(k_{b}\right)$ is the Bragg phase velocity, and $G\left(\theta_{\omega }\right)$ is the directional distribution function of the Bragg waves. $c\left(k_{b}\right)$ and $G\left(\theta_{w}\right)$ are defined as [49]

(20) $$c\left(k_{b}\right)=\sqrt{\frac{g}{k_{b}}}$$

(21) $$G\left(\theta_{w}\right)=cos^{2n}\left(\frac{\theta_{w}}{2}\right),n\in \left(2-5\right)$$

The magnitude of the wind drift caused by the ocean surface wind field can be approximated as the product of the wind drift empirical coefficient and the wind speed at 10 m above the ocean surface [40]. The wind drift velocity along the radar look direction is then given by

(22) $$v_{drift}\left(\theta_{w}\right)=\alpha_{drift}U_{10}cos\theta_{w}$$

where $\alpha_{drift}$ is the wind drift empirical coefficient, commonly taken as 3% [40,51,52]. The value of this coefficient varies across different studies but typically falls within the range of 3% ± 1% [49,53,54]. Unless otherwise specified, we set it to 3% in this study.

#### 2.4.2. Breaking Wave Velocity

Kudryavtsev et al. suggest that the Doppler velocity $v_{wb}$ of breaking wave facets can be assumed to be proportional to the weighted mean phase velocity ${\bar{c}}_{wb}$ of breaking waves within the breaking region [25]. Similar to the Bragg wave phase velocity, the directional distribution of the Doppler velocity of breaking waves is also related to the directional distribution function $G\left(\theta_{w}\right)$, i.e.,

(23) $$v_{wb}\left(\theta_{w}\right)=\varepsilon_{np}{\bar{c}}_{wb}\left[\frac{G\left(\theta_{\omega }\right)-G\left(\theta_{\omega }+\pi \right)}{G\left(\theta_{\omega }\right)+G\left(\theta_{\omega }+\pi \right)}\right]$$

(24) $${\bar{c}}_{wb}=2c\left(k_{np}\right)$$

where $k_{np}$ is the wavenumber of small-scale breaking waves, with a value of $k_{e}/10$, and $k_{e}$ is the wavenumber of the incident electromagnetic wave; the detailed derivation of ${\bar{c}}_{wb}$ can be referred to in [25]. $\varepsilon_{np}$ is related to the radar incidence angle θ, and can be calculated by

(25) $$\varepsilon_{np}=1-0.5exp\left[-\left(\theta -20^{{}^{\circ}}\right)/20^{{}^{\circ}}\right]$$

## 3. Results and Validation

### 3.1. Simulation Results of the Dynamic Ocean Surface and Backscattering

**Figure 1:**
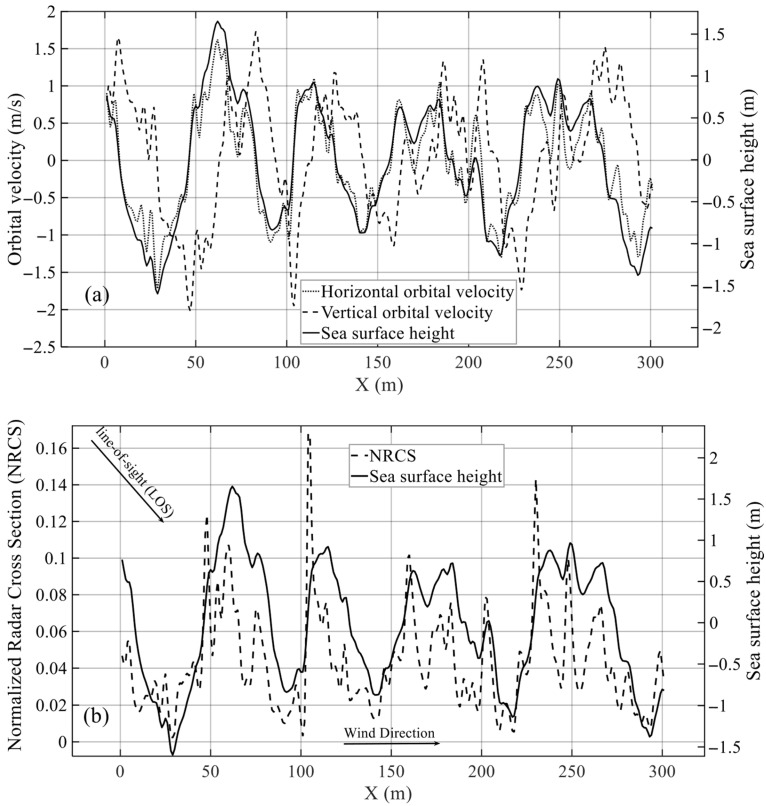
Profiles of simulated ocean surface elevation, orbital velocity, and normalized radar backscattering cross section (NRCS). (**a**) Orbital velocity and ocean surface elevation. (**b**) NRCS and ocean surface elevation. In (**a**), the solid, dotted, and dashed lines represent sea surface elevation, horizontal orbital velocity, and vertical orbital velocity, respectively. In (**b**), the solid and dashed lines represent sea surface elevation and NRCS, respectively. Simulation conditions: the radar incidence angle is 40°, the wind speed is 10 m/s, the wind direction is along the positive x-axis, the radar look direction is along the positive x-axis, and the polarization is VV.

In the simulation, the x direction is defined as the radar range direction and the y direction as the azimuth direction. Based on the ocean surface velocity potential, the ocean surface elevation field and the corresponding orbital velocity field are constructed. When the wind speed is 10 m/s and the wind direction is along the positive x direction, the profiles of ocean surface elevation and orbital velocity are shown in Figure 1a. Near the wave crests and troughs, water particles have the maximum horizontal velocity (along the wave propagation direction) and nearly zero vertical velocity (perpendicular to the wave propagation direction), whereas near the ocean surface elevation $\eta_{L}=0$, water particles have the maximum vertical velocity and nearly zero horizontal velocity. These results are consistent with the fundamental theory of ocean waves [27].

The spatial distribution of ocean surface radar backscattering is affected by tilt modulation and hydrodynamic modulation. Under the combined effects of both, wave facets tilted toward the radar usually produce stronger backscattering intensity, whereas wave facets tilted away from the radar show the opposite behavior [37]. Figure 1b presents the simulated profiles of ocean surface elevation and backscattering cross section. It can be seen that the backscattering intensity generated when wave facets are tilted toward the radar is clearly stronger than that when they are tilted away from the radar, which is consistent with the above characteristics.

### 3.2. Simulation Results of Doppler Shift Induced by Ocean Surface Motion

**Figure 2:**
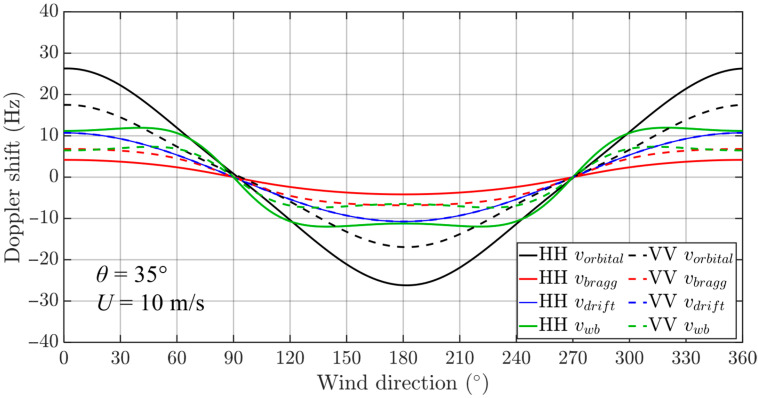
Doppler shifts in different components of ocean surface motion under HH and VV polarizations. In the figure, solid and dashed lines represent HH and VV polarization, respectively. The black, red, blue and green lines denote orbital velocity, Bragg wave phase velocity, wind drift velocity, and breaking wave velocity, respectively. Simulation conditions: the radar incidence angle is 35°, the wind speed is 10 m/s, the wind direction is from 0° to 360°, and the radar frequency is 5.36 GHz.

Based on the velocity models of different ocean surface motion components described in (7), (19), (22), and (23), we simulated the Doppler shifts in each ocean surface motion component in the C-band under HH and VV polarizations, as shown in Figure 2, in which the C-band frequency is selected as 5.36 GHz. In the simulation, the Doppler shift is defined as positive when water particles move toward the radar and negative when they move away. It can be seen from the figure that the Doppler shifts in all motion components are sensitive to wind direction and approximately follow a cosine-like pattern: the Doppler shift reaches its positive maximum in the upwind direction (wind directions 0° and 360°), approaches zero in the crosswind direction (wind directions 90° and 270°), and reaches its negative maximum in the downwind direction (wind direction 180°). Notably, the Doppler shift in breaking waves shows a different variation pattern with wind direction. It reaches its positive maximum around wind directions 50° and 310°, and its negative maximum around 130° and 230°. This may be due to the contribution of backscatter from breaking waves reaching its maximum proportion in the total radar cross section in these wind directions. In addition, the Doppler shifts in orbital velocity and breaking wave velocity are significantly affected by the polarization. Under HH polarization, the Doppler shifts in both components are notably larger than those under VV polarization, which is because the radar signal in HH polarization is more sensitive to wave breaking and the tilt modulation of large-scale waves. For Bragg wave phase velocity, the Doppler shift under HH polarization is clearly smaller than under VV polarization, since the proportion of Bragg scattering in the total radar cross section is significantly lower for HH polarization than for VV polarization. The differences in Doppler shifts in orbital velocity, breaking wave velocity, and Bragg wave phase velocity under different polarizations are consistent with the results reported in [25]. Furthermore, it can be seen that the Doppler shift caused by wind drift velocity is independent of polarization, which agrees with the findings in [52].

### 3.3. Validation of C-Band Radar WDS Simulation

**Figure 3:**
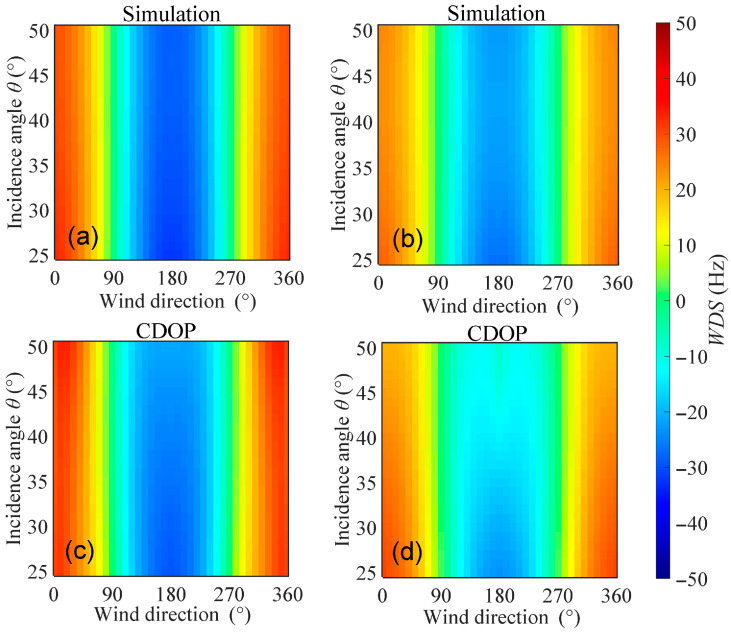
Distributions of WDS calculated by the proposed model and the CDOP model with respect to wind direction and radar incidence angle. (**a**,**b**) Proposed model; (**c**,**d**) CDOP model. (**a**,**c**) HH polarization; (**b**,**d**) VV polarization. Simulation conditions: the wind speed is 10 m/s, the radar incidence angle is from 25° to 50°, and the wind direction is from 0° to 360°.

**Figure 4:**
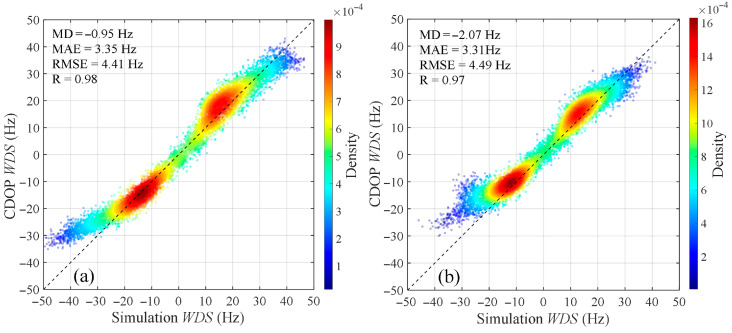
Comparison scatter plots of WDS calculated by the proposed model (horizontal axis) and the CDOP model (vertical axis). (**a**) HH polarization, (**b**) VV polarization. Simulation conditions: the radar incidence angle is from 25° to 50°, the wind direction is from 0° to 360°, and the wind speed is from 3 m/s to 13 m/s.

Using the model proposed in this study, the Doppler shift in C-band radar echoes caused by wind and waves under HH and VV polarizations is simulated and compared with the results of the CDOP model for validation. Figure 3 shows the distributions of WDS with wind direction and incidence angle when the wind speed is 10 m/s, calculated using both the proposed model and the CDOP model. It can be seen from the figure that the amplitude ranges and variation trends of the WDS from the proposed model are generally consistent with those of the CDOP model. Around the wind direction of 180°, the WDS exhibits a clear symmetric distribution, with larger Doppler shifts appearing near upwind (0° and 360°) and downwind (180°) directions. In addition, within the ranges of radar incidence angles from 25° to 50°, wind directions from 0° to 360°, and wind speeds from 3 m/s to 13 m/s, Figure 4 shows the scatter plots comparing the WDS calculated by the proposed model and the CDOP model, with color indicating the density of the points. It can be seen that the points are generally distributed along the diagonal line, and the high-density regions are clustered around the diagonal. The scatter plot results indicate that for HH polarization, the mean deviation (MD), mean absolute error (MAE), and root-mean-square error (RMSE) between the proposed model and the CDOP model are −0.95 Hz, 3.35 Hz, and 4.41 Hz, corresponding to Doppler velocity magnitudes of 0.03 m/s, 0.09 m/s, and 0.12 m/s, respectively. For VV polarization, the MD, MAE, and RMSE are −2.07 Hz, 3.31 Hz, and 4.49 Hz, corresponding to Doppler velocity magnitudes of 0.06 m/s, 0.09 m/s, and 0.13 m/s, respectively. The correlation coefficients between the proposed model and the CDOP model are 0.98 and 0.97 for HH and VV polarizations, respectively, indicating a very strong positive correlation. The low error metrics and high correlation coefficients demonstrate that the proposed model is highly consistent with the CDOP model and can effectively predict the Doppler shift in C-band radar echoes induced by wind and waves.

## 4. Discussion

### 4.1. Effects of Different Parameters on Multi-Band WDS

Investigating the WDS characteristics across different radar frequency bands contributes to improving the accuracy of ocean current retrieval in those bands. The model proposed in this study is not only applicable to C-band radar but also holds potential for application to radars in multiple frequency bands. In this study, we further investigate the effects of different parameters on WDS under X-band, Ku-band, and Ka-band conditions.

#### 4.1.1. Effects of Radar Incidence Angle on Multi-Band WDS

**Figure 5:**
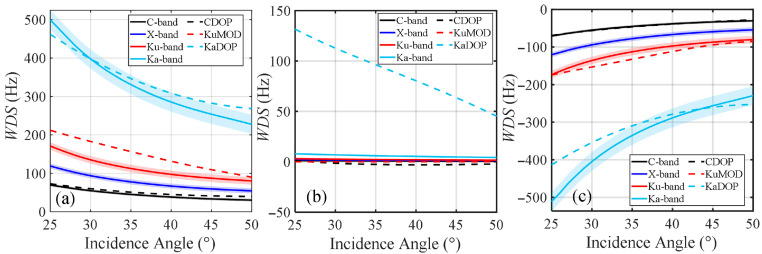
Variation in WDS with radar incidence angle for different frequency bands, calculated by the proposed model, the CDOP model, the KaDOP model and the KuMOD model. In the figure, the black, blue, red, and light blue solid lines represent C-, X-, Ku-, and Ka-band results calculated by the proposed model. The black, light blue, and red dashed lines represent the CDOP, KaDOP, and KuMOD model results, respectively. (**a**–**c**) Correspond to upwind, crosswind, and downwind directions. Simulation conditions: the radar incidence angle is from 25° to 50°, the wind speed is 10 m/s, and the polarization is HH. The confidence interval corresponds to the wind drift coefficient varying from 2% to 4% of *U*_10_.

As one of the important radar parameters, the incidence angle can significantly affect the radar observation of Doppler shift, thereby influencing the accuracy of ocean surface current retrieval. In this study, the variation in WDS with radar incidence angle was simulated for C-band, X-band, Ku-band, and Ka-band radars, and the results are shown in Figure 5, in which the frequencies of the C-, X-, Ku-, and Ka-band are selected as 5.36, 10.0, 14.6, and 37.5 GHz, respectively. Different curves represent the results calculated by the proposed model for different radar bands, as well as the results from the CDOP model, KaDOP model and KuMOD model. The results indicate that as the radar incidence angle increases from 25° to 50°, the absolute value of WDS gradually decreases. When the radar observes upwind, the ocean surface motion induced by wind and waves is toward the radar, resulting in positive Doppler shift, whereas downwind observation produces the opposite effect. Under crosswind direction, the results from both the proposed model and the CDOP model are nearly zero, while the KaDOP model still produces relatively large values of approximately 50 Hz to 140 Hz. This discrepancy arises because, in reality, wind direction is not always aligned with the dominant wave propagation direction. Consequently, even when the wind direction is perpendicular to the radar look direction, waves may still exhibit a significant Doppler velocity component along the radar look direction—an effect not accounted for in the proposed model or the CDOP model.

It can be found that the Doppler shift in ocean surface echoes is related to the frequency band of the incident electromagnetic wave. As shown in Figure 5, the WDS gradually increases from the C-band to the Ka-band, with the Ka-band results being significantly higher than those of the other three bands. This is because, as the frequency band increases, the radar can resolve shorter wavelengths of small-scale ocean surface waves, making it more sensitive to detecting small-scale Bragg waves on the ocean surface. In addition, from the downwind results in Figure 5, it can be observed that for radar incidence angles of 25° to 30°, the simulated Ka-band results from the proposed model are significantly higher than those from the KaDOP model. This difference may be attributed to the different modulation transfer functions used in the two models: the KaDOP model is based on an empirical modulation transfer function [55], whereas the proposed model uses a theoretical modulation transfer function, resulting in a higher calculated wave modulation effect in the proposed model. These results indicate that when using Ka-band radar for ocean surface current fields detection, it is particularly important to accurately estimate the WDS.

#### 4.1.2. Effects of Wind Field on Multi-Band WDS

**Figure 6:**
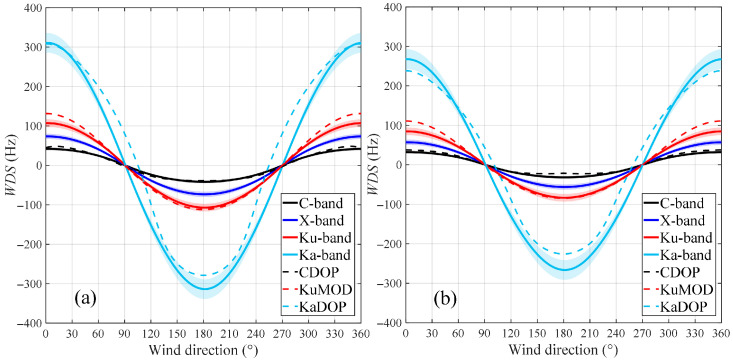
Variation in WDS with wind direction for different frequency bands, calculated by the proposed model, the CDOP model, the KaDOP model and the KuMOD model. In the figure, the black, blue, red, and light blue solid lines represent the C-, X-, Ku-, and Ka-band results calculated by the proposed model, respectively. The black, light blue, and red dashed lines represent the CDOP, KaDOP, and KuMOD model results, respectively. (**a**) HH polarization, (**b**) VV polarization. Simulation conditions: the radar incidence angle is 40°, the wind direction is from 0° to 360°, and the wind speed is 10 m/s. The confidence interval corresponds to the wind drift coefficient varying from 2% to 4% of *U*_10_.

As an important sea state parameter, both the magnitude and direction of the wind field have a significant impact on the observation of Doppler shift. In this study, we first simulated the variation in the WDS with wind direction for different frequency bands, as shown in Figure 6, where (a) corresponds to HH polarization and (b) to VV polarization. The WDS in all frequency bands shows an approximately cosine variation with wind direction: the WDS reaches the positive maximum in upwind direction (wind directions 0° and 360°); it approaches zero in crosswind direction (wind directions 90° and 270°), where the wind and wave-induced velocity on the ocean surface has almost no component in the radar look direction; and it reaches the negative maximum in downwind direction (wind direction 180°). In addition, under crosswind direction, the WDS estimated by the KaDOP model shows significant differences compared with the proposed model, similar to the analysis in Section 4.1.1. This is because the KaDOP model also accounts for deviations between the wind direction and the main wave propagation direction. The WDS values differ significantly among different radar frequency bands. The maximum WDS in the X-band and the Ku-band is approximately 1.5 times and 2.2 times that in the C-band, respectively, while the WDS in the Ka-band is much larger than that in the other three bands. By comparing the results for HH and VV polarizations, it can be found that the WDS under HH polarization is consistently larger than that under VV polarization. This is because, under the same conditions, the Doppler shifts associated with the orbital velocity and breaking wave velocity are significantly larger for HH polarization than for VV polarization. Therefore, to obtain accurate ocean surface current velocities, VV polarization should be preferentially selected across different radar frequency bands to reduce the interference of WDS on ocean current retrieval.

**Figure 7:**
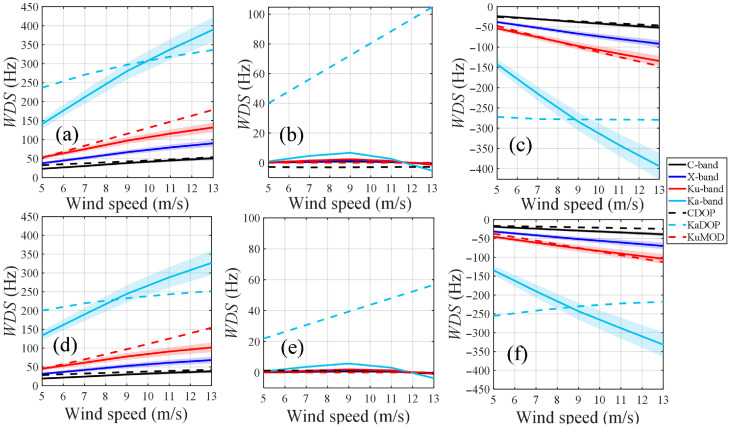
Variation in the WDS with wind speed for different frequency bands, calculated by the proposed model, the CDOP model, the KaDOP model and the KuMOD model. In the figure, the black, blue, red, and light blue solid lines represent the C-, X-, Ku-, and Ka-band results calculated by the proposed model, respectively. The black, light blue, and red dashed lines represent the CDOP, KaDOP, and KuMOD model results, respectively. (**a**–**c**) HH polarization, (**d**–**f**) VV polarization. (**a**,**d**), (**b**,**e**), and (**c**,**f**) correspond to upwind, crosswind, and downwind directions, respectively. Simulation conditions: the radar incidence angle is 40°, and the wind speed is from 5 m/s to 13 m/s. The confidence interval corresponds to the wind drift coefficient varying from 2% to 4% of *U*_10_.

In this study, we also simulated the variation in the WDS with wind speed for different frequency bands, as shown in Figure 7. Except for crosswind direction, the WDS for each band is sensitive to the magnitude of the wind speed. Specifically, under upwind direction, the WDS values for all bands are positive and increase with rising wind speed. Under downwind direction, these values are negative, and their absolute values increase with wind speed. However, the results from the KaDOP model remain almost unchanged under downwind direction. This anomalous phenomenon may be related to the fact that the observation platform used for the KaDOP model is located in a coastal shallow-water area, and its dataset has limited spatial coverage. Under crosswind direction, the WDS values for all bands are near zero, with the Ka-band results slightly higher. In contrast, the results from the KaDOP model continuously increase with wind speed, as it accounts for the component of the wave Doppler velocity along the radar look direction increasing with the increase in wind speed. Furthermore, we found that the WDS under both polarization modes exhibits similar sensitivity to wind speed, with basically the same variation patterns.

#### 4.1.3. Quantitative Analysis of the Effects of Different Parameters on Multi-Band WDS

To provide a more intuitive comparison of WDS under different conditions, the results in Figure 5, Figure 6 and Figure 7 are quantitatively summarized, and the simulated WDS values under different incidence angles, wind direction angles, and wind speeds, together with the results of the corresponding reference models for each frequency band, are presented in Table 2, Table 3 and Table 4. In Table 2, Table 3 and Table 4, “±” denotes the range of WDS uncertainty caused by a ±1% variation around the 3% reference value of the wind drift empirical coefficient.

The results indicate that the WDS for all frequency bands decreases significantly with increasing radar incidence angle. Taking the upwind simulation results of the C-band in Table 2 as an example, when the incidence angles are 25° and 50°, the corresponding WDS values are 69.71 and 30.37 Hz, respectively, representing a decrease of approximately 56%. In addition, at different incidence angles, the WDS uncertainty caused by variations in the wind drift empirical coefficient remains constant, indicating that this uncertainty is independent of the radar incidence angle and that its magnitude is identical for the upwind and downwind directions. As the wind direction angle increases from 0° to 180°, the absolute WDS values for each frequency band show a trend of first decreasing and then increasing. Taking the simulation results of the Ku-band in Table 3 as an example, under HH polarization, when the wind direction angles are 0°, 60°, 120°, and 180°, the absolute WDS values are 107.18, 57.50, 53.74, and 106.94 Hz, respectively. Comparing the WDS values across different frequency bands indicates that the WDS under HH polarization is consistently and significantly larger than that under VV polarization, which is consistent with the conclusion in Section 4.1.2. Furthermore, the WDS uncertainty caused by variations in the wind drift empirical coefficient shows a strong dependence on the wind direction angle. Specifically, this uncertainty reaches its maximum at wind direction angles of 0° and 180°, whereas at 60° and 120° it is identical and equal to half of that at 0° and 180°. This is because the wind drift velocity induced by the wind field, as determined by Equation (22), follows a cosine function with respect to the wind direction angle. Taking the Ku-band results in Table 3 as another example, the WDS uncertainty caused by variations in the wind drift empirical coefficient is ±9.74 Hz at wind direction angles of 0° and 180°, and it is reduced by half to 4.87 Hz at angles of 60° and 120°.

As described in Section 4.1.2, except for the crosswind direction, WDS increases with increasing wind speed. The simulated WDS values for different frequency bands at wind speeds of 5, 9, and 13 m/s, together with the corresponding empirical model results, are presented in Table 4. Taking the upwind results of the Ka-band in Table 4 as an example, when the wind speeds are 5, 9, and 13 m/s, the corresponding WDS values are 141.15, 281.23, and 389.85 Hz, respectively. Furthermore, the WDS uncertainty caused by variations in the wind drift empirical coefficient shows a linear relationship with wind speed. Taking the upwind case of the Ka-band as an example, when the wind speeds are 5, 9, and 13 m/s, the corresponding WDS uncertainties are 12.51, 22.52, and 32.52 Hz, respectively; the ratio of this uncertainty to the wind speed is approximately constant at 2.5. This linear characteristic is likewise determined by Equation (22).

### 4.2. Comparison of the Proposed Model with the KaDOP and KuMOD Models

As described in Section 4.1, the model proposed is not only applicable to C-band radar but also holds potential for application to various radar bands. The effectiveness of the proposed model in the C-band has been validated in Section 3.3. To further evaluate the proposed model’s performance in the Ka-band and Ku-band, its results were compared with those from the KaDOP model and the KuMOD model, respectively.

**Figure 8:**
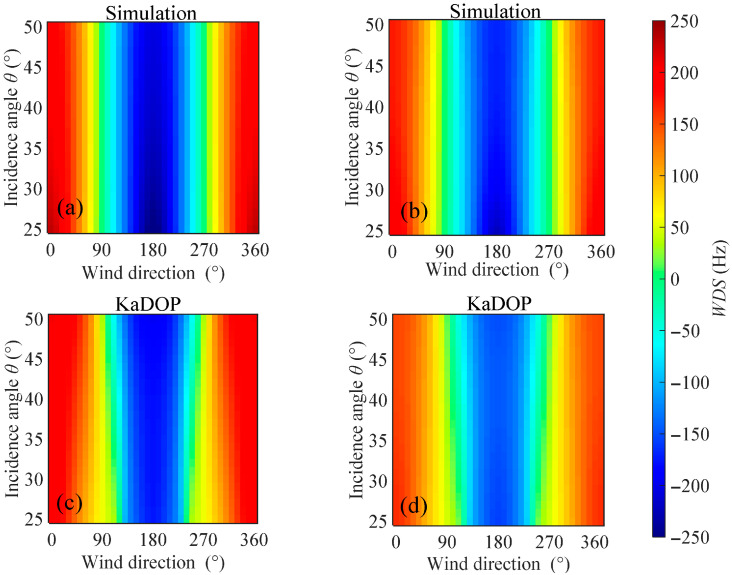
Distributions of WDS calculated by the proposed model and the KaDOP model with respect to wind direction and radar incidence angle. (**a**,**b**) Proposed model; (**c**,**d**) KaDOP model. (**a**,**c**) HH polarization; (**b**,**d**) VV polarization. Simulation conditions: the wind speed is 10 m/s, the radar incidence angle is from 25° to 50°, and the wind direction is from 0° to 360°.

**Figure 9:**
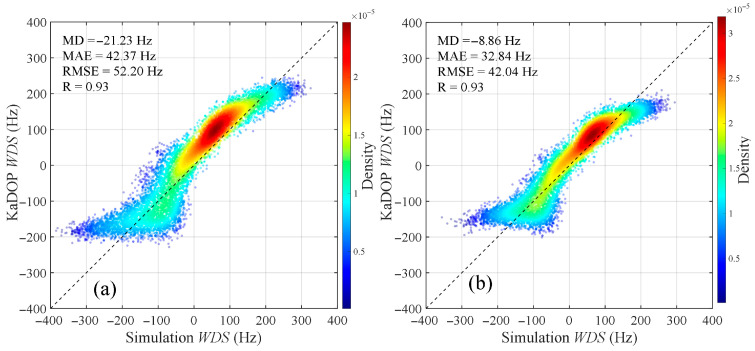
Comparison scatter plots of WDS calculated by the proposed model (horizontal axis) and the KaDOP model (vertical axis). (**a**) HH polarization, (**b**) VV polarization. Simulation conditions: the radar incidence angle is from 25° to 50°, the wind direction is from 0° to 360°, and the wind speed is from 3 m/s to 13 m/s.

In the Ka-band, the distributions of WDS with wind direction and incidence angle were calculated using both the model proposed in this study and the KaDOP model at a wind speed of 10 m/s, with the results shown in Figure 8, respectively. It can be observed that the amplitude range and variation trend of WDS calculated by the proposed model are generally consistent with those calculated by the KaDOP model. On both sides of the wind direction angle of 180°, the WDS exhibits a clear symmetric distribution, with larger Doppler shifts appearing near upwind (0° and 360°) and downwind (180°) directions. Additionally, within the ranges of radar incidence angles from 25° to 50°, wind directions from 0° to 360°, and wind speeds from 3 m/s to 13 m/s, scatter plots comparing the WDS calculated by the proposed model and the KaDOP model are shown in Figure 9, with color depth indicating the density of the points. It can be seen that the points are generally distributed along the diagonal line, with the high-density regions concentrated near the diagonal. The scatter plot results indicate that for HH polarization, the MD, MAE, and RMSE between the proposed model and the KaDOP model are −21.23 Hz, 42.37 Hz, and 52.20 Hz, corresponding to Doppler velocity magnitudes of 0.08 m/s, 0.17 m/s, and 0.21 m/s, respectively. For VV polarization, the MD, MAE, and RMSE are −8.86 Hz, 32.84 Hz, and 42.04 Hz, corresponding to Doppler velocity magnitudes of 0.04 m/s, 0.13 m/s, and 0.17 m/s, respectively. The correlation coefficients between the proposed model and the KaDOP model for both polarization modes are 0.93, indicating a strong positive correlation. The low error metrics and high correlation coefficients demonstrate that the proposed model has good applicability in the Ka-band.

**Figure 10:**
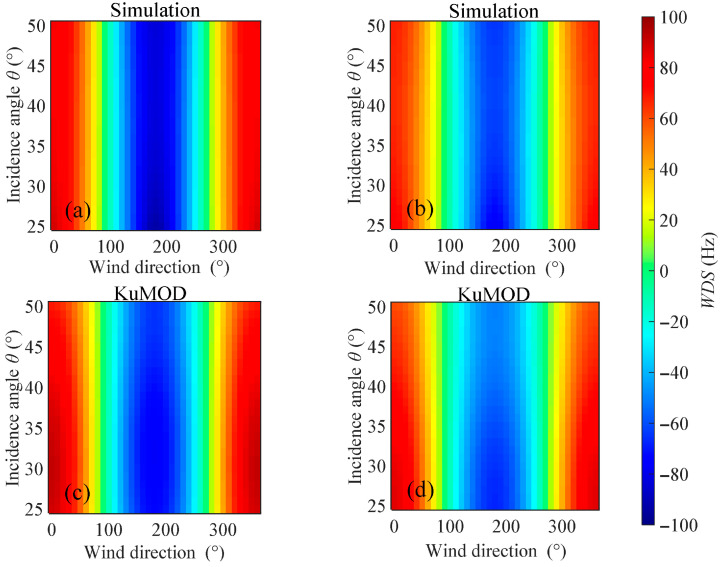
Distributions of WDS calculated by the proposed model and the KuMOD model with respect to wind direction and radar incidence angle. (**a**,**b**) Proposed model; (**c**,**d**) KuMOD model. (**a**,**c**) HH polarization; (**b**,**d**) VV polarization. Simulation conditions: the wind speed is 10 m/s, the radar incidence angle is from 25° to 50°, and the wind direction is from 0° to 360°.

**Figure 11:**
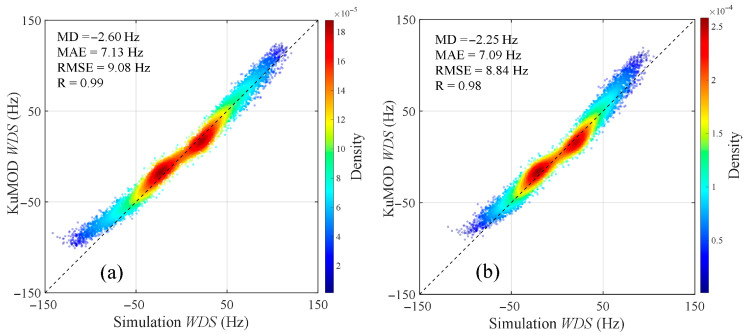
Comparison scatter plots of WDS calculated by the proposed model (horizontal axis) and the KuMOD model (vertical axis). (**a**) HH polarization, (**b**) VV polarization. Simulation conditions: the radar incidence angle is from 25° to 50°, the wind direction is from 0° to 360°, and the wind speed is from 3 m/s to 13 m/s.

In the Ku-band, the distributions of WDS with wind direction and incidence angle were calculated using the model proposed in this study and the KuMOD model, respectively, at a wind speed of 10 m/s, as shown in Figure 10. Similar to the analysis in the Ka-band, it can be observed from the figure that the amplitude range and variation trend of WDS calculated by the proposed model are generally consistent with those calculated by the KuMOD model. On both sides of the wind direction angle of 180°, the WDS exhibits a symmetric distribution, with larger Doppler shifts also appearing near upwind (0° and 360°) and downwind (180°) directions. Additionally, within the ranges of radar incidence angles from 25° to 50°, wind directions from 0° to 360°, and wind speeds from 3 m/s to 13 m/s, scatter plots comparing the WDS calculated by the proposed model and the KuMOD model are presented in Figure 11. It can be seen that the points are generally distributed along the diagonal line, with the high-density regions concentrated near the diagonal. The scatter plot results indicate that for HH polarization, the MD, MAE, and RMSE between the proposed model and the KuMOD model are −2.60 Hz, 7.13 Hz, and 9.08 Hz, corresponding to Doppler velocity magnitudes of 0.03 m/s, 0.07 m/s, and 0.09 m/s, respectively. For VV polarization, the MD, MAE, and RMSE are −2.25 Hz, 7.09 Hz, and 8.84 Hz, corresponding to Doppler velocity magnitudes of 0.02 m/s, 0.07 m/s, and 0.09 m/s, respectively. The correlation coefficients between the proposed model and the KuMOD model are 0.99 and 0.98 for HH and VV polarizations, respectively, indicating a very strong positive correlation. The low error metrics and high correlation coefficients demonstrate that the proposed model is highly consistent with the KuMOD model and can effectively predict the Doppler shift in Ku-band radar echoes induced by wind and wave.

### 4.3. Application of the Proposed Model to Ocean Current Inversion

Currently, Sentinel-1 data are publicly available and measured ocean current data that are spatiotemporally matched with them can be obtained for comparative validation, whereas SAR data of other bands that match measured current data are relatively difficult to acquire. Therefore, to evaluate the applicability of the proposed model to SAR-based ocean current retrieval, the model is applied to C-band Sentinel-1 OCN data for current inversion. The retrieved currents are then validated through comparison with temporally and spatially collocated high-frequency (HF) radar current measurements along the East and West Coasts of the United States.

In this study, seven scenes of temporally and spatially matched Sentinel-1 OCN data and HF radar ocean current data along the East Coast of the United States were acquired from 19 September 2023 to 29 December 2023, and five scenes of corresponding data along the West Coast of the United States were acquired from 25 December 2023 to 10 June 2024. The matching criteria were as follows: spatial overlap and a time interval of no more than 1 h. The temporally and spatially matched HF radar data obtained in this study have a temporal resolution of 1 h and a spatial resolution of 6 km. Based on the 1 min step used in temporal interpolation and the 1 km spatial resolution of the OCN data, the HF radar data were interpolated to match the resolution of the SAR data. The OCN data provides radar parameters, measured Doppler centroid frequency shift, predicted Doppler frequency shift caused by the relative motion between the satellite and the Earth, and ocean surface wind field parameters. First, the predicted Doppler frequency shift is removed from the measured Doppler centroid frequency shift. Then, using the method proposed by Yang [23], electromagnetic pointing errors are corrected, and the total Doppler shift caused by wind and waves as well as ocean currents is obtained. Next, based on the wind speed, wind direction, radar incidence angle, and polarization mode in the OCN data, the WDS is calculated using both the model proposed in this study and the CDOP model. This Doppler shift is then removed from the total Doppler shift, leaving only the Doppler shift corresponding to the ocean current velocity. This Doppler shift is then converted into Doppler velocity and projected onto the radar range direction, resulting in the ocean surface current velocity in the radar look direction [56].

**Figure 12:**
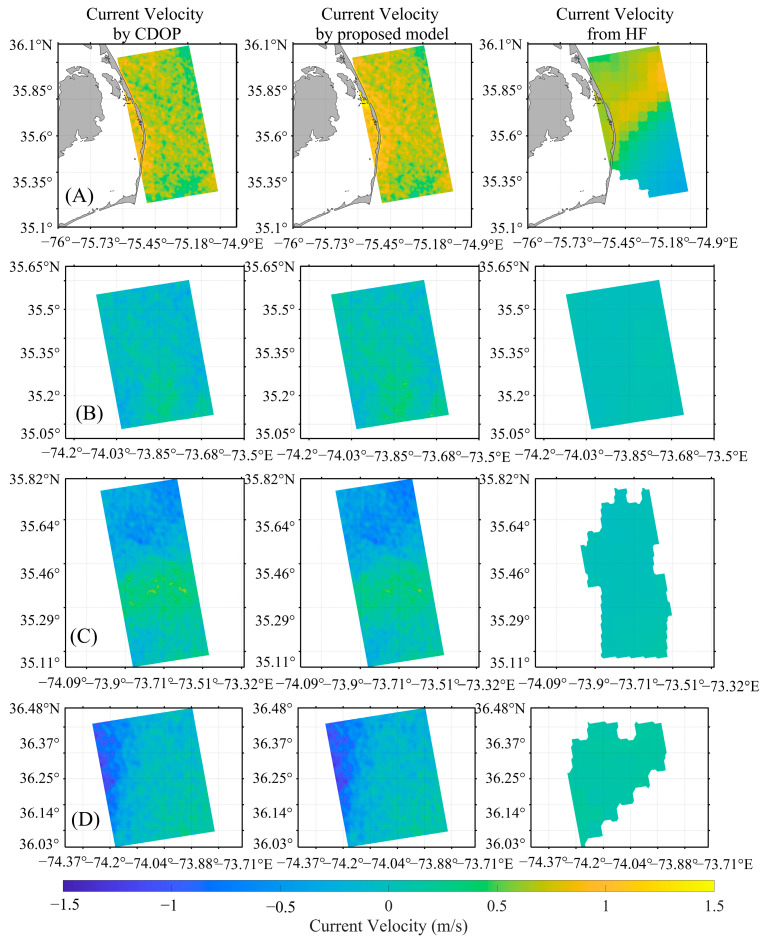
Ocean current distribution maps inverted from Sentinel-1 OCN data along the East Coast. (**A**–**D**) correspond to the four scenes listed in the first four rows of the East Coast data in Table 5. The first, second, and third columns show currents retrieved by the CDOP model, the proposed model, and currents from HF radar (projected onto the radar look direction), respectively.

**Figure 13:**
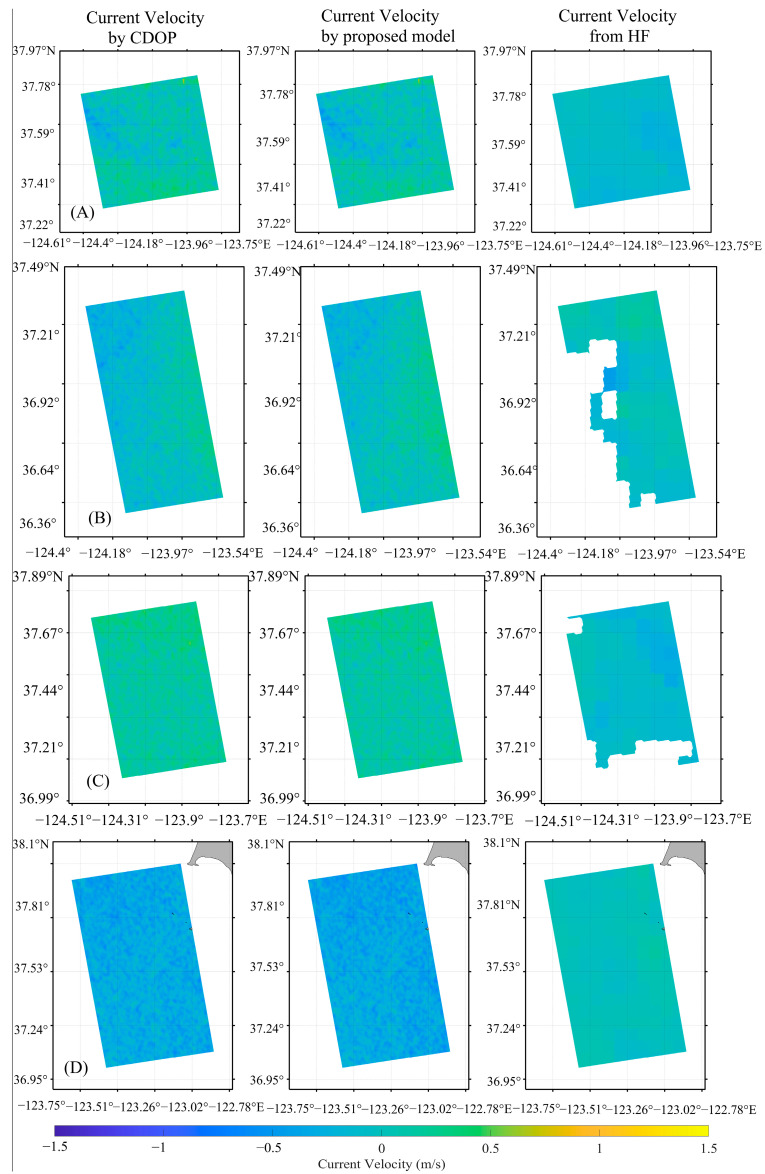
Ocean current distribution maps inverted from Sentinel-1 OCN data along the West Coast. (**A**–**D**) correspond to the four scenes listed in the first four rows of the West Coast data in Table 5. The first, second, and third columns show currents retrieved by the CDOP model, the proposed model, and currents from HF radar (projected onto the radar look direction), respectively.

Using the method described above, ocean current inversion was performed based on twelve scenes of OCN data acquired along the East and West Coasts of the United States, and the ocean current distribution maps of four of the scenes for the East Coast and four of the scenes for the West Coast are shown in Figure 12 and Figure 13, respectively (the first to fourth rows of Figure 12 correspond to the first to fourth rows of the East Coast results in Table 5, and the first to fourth rows of Figure 13 correspond to the first to fourth rows of the West Coast results in Table 5). In the figure, the first column shows the ocean surface current velocities after removing the WDS using the CDOP model; the second column shows the ocean surface current velocities after removing the WDS using the model proposed in this study; the third column shows the SAR look direction component of the HF radar current velocities. It can be observed that the ocean current magnitudes and distributions obtained using both the CDOP model and the proposed model are very similar. The errors in the comparison of the ocean currents retrieved using the CDOP model and the proposed model with the HF radar ocean current data for the twelve scenes are shown in Table 5. For the radar look direction current velocities retrieved using the CDOP model, the MD, MAE, and RMSE are −0.02 m/s, 0.25 m/s, 0.30 m/s, respectively. For the radar look direction current velocities retrieved using the proposed model, the MD, MAE, and RMSE are −0.04 m/s, 0.26 m/s, 0.32 m/s, respectively. The inversion results using the proposed model are very similar to those obtained using the CDOP model, indicating that the proposed model performs well in SAR-based ocean current inversion.

## 5. Conclusions

In SAR-based ocean surface current inversion, the WDS is a critical factor influencing accuracy. In this study, the WDS was investigated through simulation-based approaches. First, the ocean surface elevation field and the corresponding wave orbital velocity field were established based on the ocean surface velocity potential. Then, the radar backscattering coefficient of the ocean surface was simulated using the TSM, combined with a breaking wave scattering model, while accounting for tilt modulation and hydrodynamic modulation effects. Furthermore, based on the simulation of various motion velocity components of ocean surface water particles, a WDS simulation model was developed according to the Doppler shift theory of ocean surface motion proposed by Chapron. By comparing the simulated WDS with those calculated using the CDOP empirical model, we preliminarily verify the validity of the proposed model for C-band WDS. Under co-polarization conditions, the correlation coefficient between the results of the model proposed in this study and the CDOP model reaches up to 0.97, while the MD, MAE, and RMSE do not exceed −2.07 Hz, 3.35 Hz, and 4.49 Hz, respectively, corresponding to Doppler velocity magnitudes of 0.06 m/s, 0.09 m/s, and 0.13 m/s. These results demonstrate that the proposed model effectively predicts the Doppler shift in C-band radar echoes.

The simulation model proposed in this study is applicable to radar systems operating at multiple frequency bands. The effects of radar parameters and wind fields on the WDS were further investigated for the C-, X-, Ku-, and Ka-bands. The simulation results show that the WDS gradually increases with increasing radar frequency, and the WDS in the Ka-band is significantly larger than those in the other three bands. The WDS under VV polarization is consistently lower than that under HH polarization, indicating that VV polarization is more suitable for high-precision ocean current inversion. The WDS exhibits a quasi-cosine dependence on wind direction, reaching a positive maximum under upwind direction and a negative maximum under downwind direction. Except for crosswind direction, the WDS increases approximately linearly with wind speed. To further verify the applicability of the proposed model across different frequency bands, the simulated Ka-band WDSs produced by the model proposed in this study are compared with those calculated using the KaDOP model. The results show that the correlation coefficient between the two reaches 0.93, while the MD, MAE, and RMSE do not exceed −21.23 Hz, 42.37 Hz, and 52.20 Hz, respectively, corresponding to Doppler velocity magnitudes of 0.08 m/s, 0.17 m/s, and 0.21 m/s. In addition, we compared the simulated WDS of the proposed model at the Ku-band with the KuMOD model. The results show that the correlation coefficient between the two reaches 0.98, while the MD, MAE, and RMSE do not exceed −2.60 Hz, 7.13 Hz, and 9.08 Hz, respectively, corresponding to Doppler velocity magnitudes of 0.03 m/s, 0.07 m/s, and 0.09 m/s. These results demonstrate that the model proposed in this study exhibits good simulation performance in the Ka- and Ku-band.

Finally, the model proposed in this study was applied to current inversion based on Sentinel-1 OCN data. The retrieved currents were compared and validated against spatiotemporally matched HF radar current data. The comparison results show that the MD, MAE, and RMSE of the currents retrieved using the proposed model are −0.04 m/s, 0.26 m/s, and 0.32 m/s, respectively, which are comparable to the accuracy achieved by the CDOP-based inversion (with MD, MAE, and RMSE of −0.02 m/s, 0.25 m/s, and 0.30 m/s, respectively). The errors in the inversion results may be related to the limited accuracy of the wind field data used and the inherent measurement uncertainties of the HF radar. Overall, these results indicate that the proposed model exhibits good applicability in ocean current inversion.

It should be noted that although the proposed model has been validated against existing reference models and satellite measurements, it is built upon linear wave theory and does not account for swell-dominated conditions or nonlinear wave–wave interactions. In reality, ocean waves are generally nonlinear and often consist of a mixture of wind waves and swell. Therefore, future studies may incorporate nonlinear corrections and swell-related parameters to further extend the applicability of the model. In addition, the present simulations adopt a fixed wind-driven drift coefficient, for which no universally accepted value currently exists; future work may focus on its systematic estimation or optimization. Regarding multi-band validation, comparative verification with existing models has been conducted for the C, Ku, and Ka bands, while for other frequency bands, future validation will be carried out using mature models or observational data to further assess the applicability and robustness of the proposed model across different bands.

## Figures and Tables

**Table 1:** Values of input parameters for dynamic sea surface simulation.

Parameter	Value
Grid size	1.0 (m)
Fixed random seed	42 *
Initial random phase	0–2π
Wave frequency range	0.05–0.50 (Hz)
Direction angle	0–360 (°)
Inverse wave age	0.84

* The fixed random seed 42 was set using rng(42) in MATLAB R2022b.

**Table 2:** Quantitative summary of WDS under different radar incidence angles. Units: Hz.

Incidence Angle	Wind Direction	C-Band	CDOP	Ku-Band	KuMOD	Ka-Band	KaDOP
25°	upwind	69.71 ± 3.58	72.92	170.95 ± 9.74	212.03	499.42 ± 25.02	462.03
	downwind	−70.56 ± 3.58	−69.32	−173.24 ± 9.74	−174.07	−511.34 ± 25.02	−413.52
35°	upwind	45.23 ± 3.58	51.11	112.65 ± 9.74	156.60	330.95 ± 25.02	346.70
	downwind	−40.67 ± 3.58	−41.53	−102.65 ± 9.74	−119.95	−305.13 ± 25.02	−289.56
50°	upwind	30.37 ± 3.58	40.00	80.29 ± 9.74	89.93	227.42 ± 25.02	268.50
	downwind	−30.22 ± 3.58	−27.54	−80.04 ± 9.74	−85.01	−229.20 ± 25.02	−252.88

**Table 3:** Quantitative summary of WDS under different wind direction angles. Units: Hz.

Wind Direction Angle	Polarization	C-Band	CDOP	Ku-Band	KuMOD	Ka-Band	KaDOP
0°	HH	41.90 ± 3.58	45.01	107.18 ± 9.74	131.52	310.64 ± 25.02	308.55
	VV	32.24 ± 3.58	37.75	84.74 ± 9.74	110.99	267.46 ± 25.02	238.35
60°	HH	24.62 ± 1.79	24.27	57.50 ± 4.87	63.30	161.15 ± 12.51	199.96
	VV	19.01 ± 1.79	22.02	45.45 ± 4.87	52.20	139.83 ± 12.51	144.41
120°	HH	−23.26 ± 1.79	−26.83	−53.74 ± 4.87	−58.39	−151.20 ± 12.51	−97.89
	VV	−17.73 ± 1.79	−17.15	−41.89 ± 4.87	−45.62	−130.07 ± 12.51	−88.15
180°	HH	−41.73 ± 3.58	−38.71	−106.94 ± 9.74	−111.86	−313.52 ± 25.02	−278.74
	VV	−361.69 ± 3.58	−21.26	−83.37 ± 9.74	−84.67	−266.17 ± 25.02	−225.91

**Table 4:** Quantitative summary of WDS under different wind speeds. Units: Hz.

Wind Speed	Wind Direction	C-Band	CDOP	Ku-Band	KuMOD	Ka-Band	KaDOP
5 m/s	upwind	23.21 ± 1.79	32.78	53.20 ± 4.87	52.39	141.15 ± 12.51	236.67
	downwind	−23.30 ± 1.79	−25.35	−53.48 ± 4.87	−47.29	−142.43 ± 12.51	−272.06
9 m/s	upwind	38.39 ± 3.22	42.49	97.44 ± 8.77	115.73	281.23 ± 22.52	297.46
	downwind	−38.20 ± 3.22	−36.07	−97.15 ± 8.77	−99.40	−283.80 ± 22.52	−278.61
13 m/s	upwind	50.75 ± 4.65	53.58	132.14 ± 12.66	178.61	389.85 ± 32.52	336.22
	downwind	−51.50 ± 4.65	−46.44	−134.13 ± 12.66	−146.71	−393.97 ± 32.52	−279.35

**Table 5:** Comparison of ocean currents retrieved by the CDOP model and the model proposed in this study with HF radar ocean current data.

Area	Index	CDOP vs. HF (m/s) (MD, MAE, RMSE)	Proposed Model vs. HF (m/s) (MD, MAE, RMSE)
	A	0.26, 0.37, 0.45	0.34, 0.39, 0.48
	B	−0.05, 0.12, 0.15	0.05, 0.12, 0.15
	C	−0.05, 0.24, 0.29	−0.11, 0.25, 0.31
East Coast	D	−0.34, 0.35, 0.42	−0.38, 0.38, 0.45
	E	−0.06, 0.20, 0.25	−0.20, 0.27, 0.33
	F	−0.18, 0.23, 0.28	−0.22, 0.26, 0.31
	G	0.24, 0.32, 0.40	0.19, 0.32, 0.40
	A	0.17, 0.21, 0.25	0.13, 0.18, 0.22
	B	0.01, 0.14, 0.18	0.05, 0.16, 0.19
West Coast	C	0.28, 0.28, 0.31	0.26, 0.26, 0.30
	D	−0.32, 0.32, 0.34	−0.35, 0.35, 0.37
	E	−0.19, 0.21, 0.25	−0.22, 0.23, 0.27
Average Metric Value		−0.02, 0.25, 0.30	−0.04, 0.26, 0.32

## Data Availability

The data that support the findings of this study are available from the authors upon reasonable request.
